# Supplementary material for: MetaRibo-Seq measures translation in microbiomes
Source: Nat Commun. 2020 Jun 29;11:3268. doi: 10.1038/s41467-020-17081-z (PMC7324362; doi:10.1038/s41467-020-17081-z)
Supplement: Supplementary file 10 — Supplementary Data 7 [file 41467_2020_17081_MOESM10_ESM.zip › File2/Confidence_High_Taxonomy/179090_out.krona.html]

Javascript must be enabled to view this page.

members
magnitude
magnitudeUnassigned
count
unassigned
taxon
rank

179090\_out

39

2
superkingdom
39

phylum
976
39

200643
class
39

39
171549
order

39
815
family

816
genus
39


SRS140492\_contig\_number\_2745
46506
species
1

1
338188
species

SRS047044\_contig\_number\_12753

2292951
species
1

SRS016897\_contig\_number\_1255

species
47678
1

SRS047433\_contig\_number\_21501


SRS013158\_contig\_number\_1449SRS014235\_contig\_number\_15775SRS014534\_contig\_number\_1044SRS015890\_contig\_number\_814SRS016495\_contig\_number\_16086SRS019286\_contig\_number\_5410SRS021153\_contig\_number\_1137SRS023971\_contig\_number\_254SRS053214\_contig\_number\_4869SRS101268\_contig\_number\_941SRS1041130\_contig\_number\_987SRS149879\_contig\_number\_16712SRS893380\_contig\_number\_650
13
371601
species

species
818
3

SRS016267\_contig\_number\_9415SRS049402\_contig\_number\_1287SRS971276\_contig\_number\_693

28116
species
2

SRS077194\_contig\_number\_contig-100\_269.269SRS893285\_contig\_number\_606


SRS1041036\_contig\_number\_779
2292930
species
1


SRS012969\_contig\_number\_2697SRS017521\_contig\_number\_42462SRS018351\_contig\_number\_3142SRS020233\_contig\_number\_21889SRS048164\_contig\_number\_11117SRS049896\_contig\_number\_24112SRS050925\_contig\_number\_1170SRS058070\_contig\_number\_305SRS064973\_contig\_number\_1008SRS1041090\_contig\_number\_contig-100\_2517.47218SRS1041116\_contig\_number\_contig-100\_1745.53892SRS1041145\_contig\_number\_contig-100\_2558.180898SRS1041146\_contig\_number\_contig-100\_399.400SRS1054928\_contig\_number\_2318SRS142599\_contig\_number\_3458SRS144506\_contig\_number\_39256
16
2292955
species
